# Enhancing Vaccine Efficacy and Stability: A Review of the Utilization of Nanoparticles in mRNA Vaccines

**DOI:** 10.3390/biom14081036

**Published:** 2024-08-20

**Authors:** Nargish Parvin, Sang Woo Joo, Tapas Kumar Mandal

**Affiliations:** School of Mechanical Engineering, Yeungnam University, Gyeongsan 38541, Republic of Korea; nargish.parvin@gmail.com

**Keywords:** nanoparticles, mRNA vaccines, lipid nanoparticles (LNPs), vaccine delivery systems, vaccine stability

## Abstract

The development of vaccines has entered a new era with the advent of nanotechnology, particularly through the utilization of nanoparticles. This review focuses on the role of nanoparticles in enhancing the efficacy and stability of mRNA vaccines. Nanoparticles, owing to their unique properties such as high surface area, tunable size, and their ability to be functionalized, have emerged as powerful tools in vaccine development. Specifically, lipid nanoparticles (LNPs) have revolutionized the delivery of mRNA vaccines by protecting the fragile mRNA molecules and facilitating their efficient uptake by cells. This review discusses the various types of nanoparticles employed in mRNA vaccine formulations, including lipid-based, polymer-based, and inorganic nanoparticles, highlighting their advantages and limitations. Moreover, it explores the mechanisms by which nanoparticles improve immune responses, such as enhanced antigen presentation and the prolonged release of mRNA. This review also addresses the challenges and future directions in nanoparticle-based vaccine development, emphasizing the need for further research to optimize formulations for broader applications. By providing an in-depth analysis of the current advancements in and potential of nanoparticles in mRNA vaccines, this review aims to shed light on their critical role in combating infectious diseases and improving public health outcomes.

## 1. Introduction

The field of vaccine development has been revolutionized by the advent of nanotechnology, particularly through the utilization of nanoparticles. Nanoparticles possess unique physicochemical properties such as high surface area, tunable size, and the ability to be functionalized, making them powerful tools in vaccine design and delivery systems. This review explores the pivotal role of nanoparticles in enhancing the efficacy and stability of mRNA vaccines, thereby advancing their potential in combating infectious diseases and improving public health outcomes.

### 1.1. Overview of mRNA Vaccines

**Figure 1:**
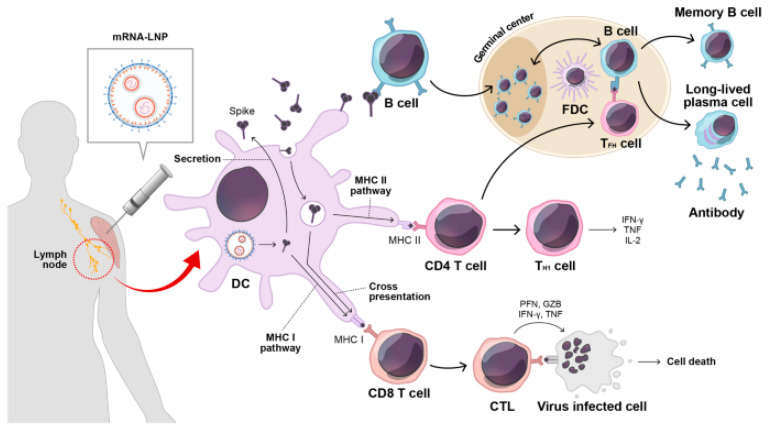
After mRNA vaccination, secreted spike antigens are identified by cognate B-cells and induce potent neutralizing antibody responses with a strong germinal center reaction. Dendritic cells (DCs) uptake soluble spike antigens and stimulate antigen-specific CD4 and CD8 T-cells via the MHC II and cross-presentation pathways, respectively. In addition, endogenously expressed spike proteins in DCs can activate antigen-specific CD8 T-cells through the MHC I pathway. LNP, lipid nanoparticle; FDC, follicular dendritic cell; TFH, T follicular helper cell; TH1, type 1 T helper cell; CTL, cytotoxic T lymphocyte; PFN, perforin; GZB, granzyme B; IFN-γ, interferon gamma; TNF-α, tumor necrosis factor-alpha. Reprinted with permission from Ref. [1].

Nanoparticles, especially lipid nanoparticles (LNPs), have emerged as key vehicles for mRNA delivery due to their ability to encapsulate and protect mRNA from enzymatic degradation and immune recognition. LNPs enhance the stability of mRNA vaccines and facilitate their cellular uptake, thereby optimizing antigen expression and immune stimulation. Figure 1 illustrates the immune response triggered by mRNA vaccination. Upon vaccination, secreted spike antigens are recognized by B-cells, leading to the production of potent neutralizing antibodies and the formation of a robust germinal center. Dendritic cells (DCs) play a crucial role by capturing soluble spike antigens and presenting them to CD4 and CD8 T-cells via MHC II and cross-presentation pathways, respectively. Additionally, DCs expressing spike proteins can activate CD8 T-cells through the MHC I pathway. This comprehensive immune activation involves various components, including lipid nanoparticles (LNPs) and immune cells such as follicular dendritic cells (FDCs) and T follicular helper cells (TFH), contributing to a coordinated immune defense involving cytotoxic T lymphocytes (CTLs) and other key factors [1]. Recent studies have demonstrated that LNPs can be engineered to enhance the pharmacokinetics and biodistribution of mRNA vaccines, improving their overall efficacy and safety profiles [2].

Messenger RNA (mRNA) vaccines represent a revolutionary approach in the field of vaccinology, providing significant advantages over traditional vaccine platforms such as live-attenuated or inactivated vaccines. These vaccines utilize synthetic mRNA to encode the antigen of interest, which, when introduced into the host cells, is translated into the corresponding protein antigen. This antigen is then recognized by the immune system, thereby eliciting an immune response [3].

The concept of using mRNA as a therapeutic tool was initially met with skepticism due to its inherent instability and the challenges associated with its delivery. However, advancements in mRNA synthesis and delivery technologies have addressed many of these challenges, leading to the successful development of mRNA vaccines. The COVID-19 pandemic accelerated the development and approval of the first mRNA vaccines, namely Pfizer-BioNTech’s BNT162b2 and Moderna’s mRNA-1273, both of which demonstrated high efficacy and safety in clinical trials [4,5].

Key advantages of mRNA vaccines:

1. **Rapid Development and Manufacturing**: Unlike conventional vaccines, which require the cultivation of viruses or bacteria, mRNA vaccines can be synthesized quickly and in large quantities using cell-free processes. This rapid production capability is crucial for responding to emerging infectious diseases [6].
2. **Safety**: mRNA vaccines do not contain live pathogens, eliminating the risk of infection. Additionally, mRNA does not integrate into the host genome, reducing the potential for long-term genetic effects [7].
3. **Versatility**: mRNA vaccines can be designed to target a wide range of infectious diseases and cancers. The same production platform can be adapted to produce different mRNA sequences, facilitating the development of vaccines against new or multiple targets [8].

### 1.2. Importance of Vaccine Efficacy and Stability

The efficacy and stability of vaccines are critical determinants of their successful deployment and public health impact. mRNA vaccines, while promising, are inherently unstable and susceptible to degradation, posing challenges for storage and distribution. Nanoparticles address these challenges by providing a protective environment for mRNA, ensuring its integrity during storage and transit.

In addition to LNPs, polymer-based nanoparticles offer alternative platforms for mRNA vaccine delivery, leveraging biocompatible and biodegradable materials to enhance payload protection and controlled release kinetics. These nanoparticles can be engineered to optimize mRNA stability and bioavailability, thereby augmenting vaccine efficacy through tailored delivery strategies [9].

Furthermore, inorganic nanoparticles such as gold nanoparticles have shown promise in enhancing antigen presentation and immune modulation, thereby augmenting the immunogenicity of mRNA vaccines. Their unique physicochemical properties enable precise control over vaccine formulation and delivery parameters, paving the way for next-generation vaccine platforms [10].

The efficacy of a vaccine is determined by its ability to induce a robust and durable immune response that protects against the target disease. For mRNA vaccines, this involves the successful delivery of mRNA into host cells, the efficient translation of the mRNA into the target protein, and the presentation of this protein to the immune system to elicit both humoral and cellular immunity [11].

Stability is a critical factor influencing the efficacy of mRNA vaccines. mRNA is inherently unstable due to its susceptibility to enzymatic degradation and hydrolysis. To address this, various strategies have been employed to enhance mRNA stability:

1. **Chemical Modifications**: The incorporation of modified nucleosides, such as pseudouridine, can enhance mRNA stability and translation efficiency while reducing immunogenicity [12].
2. **Optimization of mRNA Sequence**: Codon optimization and the inclusion of untranslated regions (UTRs) can improve mRNA stability and translation [13].
3. **Encapsulation in Nanoparticles**: Nanoparticles protect mRNA from degradation and facilitate its delivery into host cells. Lipid nanoparticles (LNPs) are the most commonly used delivery systems for mRNA vaccines, providing a stable and efficient means of delivering mRNA into the cytoplasm of target cells [14].
4. **Vaccine efficacy and stability are interconnected:** An unstable mRNA vaccine may degrade before it reaches the target cells, resulting in lower protein expression and a weaker immune response.

**Figure 2:**
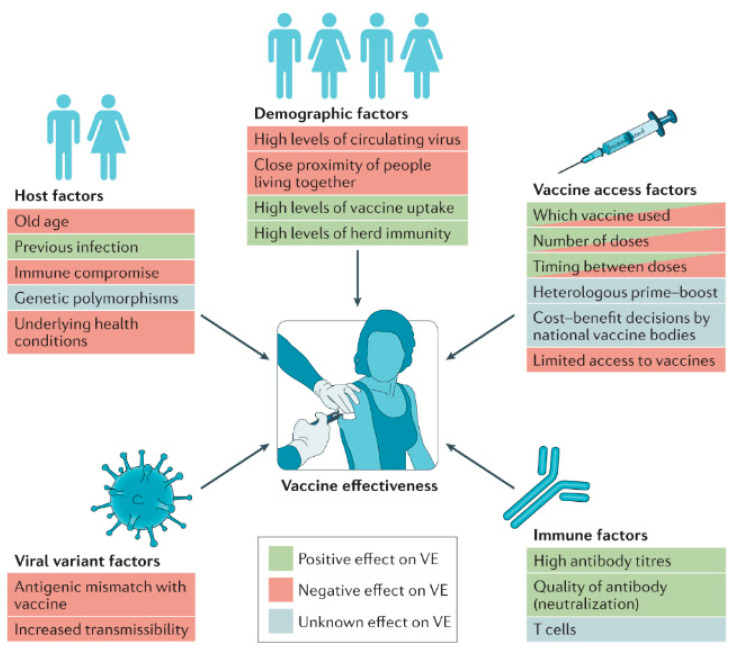
Determinants of vaccine efficacy. Various factors can enhance or reduce vaccine efficacy (VE) at the individual level and across the broader population. Reprinted with permission from Ref. [15].

Figure 2 depicts the key determinants influencing vaccine efficacy (VE) at both the individual and population levels. Factors that can enhance vaccine efficacy include the quality of the vaccine formulation, optimal dosing schedules, and timely administration. On the other hand, factors that might reduce efficacy encompass issues like variations in immune response among individuals, vaccine storage conditions, and potential interference from pre-existing immunity. Understanding these determinants helps in optimizing vaccine design and implementation strategies to ensure maximal effectiveness across diverse populations [15]. Conversely, a stable and efficiently delivered mRNA vaccine can induce a strong and lasting immune response, providing effective protection against the target disease [16].

The clinical success of mRNA vaccines during the COVID-19 pandemic has underscored the importance of optimizing both efficacy and stability. Pfizer-BioNTech and Moderna’s vaccines demonstrated high efficacy rates of approximately 95% and 94%, respectively, in preventing symptomatic COVID-19 infection [4,5]. These vaccines also showed good stability profiles, although they required storage at very low temperatures to maintain their efficacy over time.

Ongoing research aims to further enhance the stability of mRNA vaccines to improve their practicality and accessibility. Innovations such as lyophilization (freeze-drying) and the development of more stable nanoparticle formulations are being explored to enable mRNA vaccines to be stored and transported at higher temperatures [17]. So, the development and deployment of mRNA vaccines represent a significant advancement in vaccine technology, driven by the need for rapid, safe, and versatile vaccine platforms. The optimization of mRNA stability and delivery systems is crucial for maximizing vaccine efficacy, ensuring that these innovative vaccines can provide robust and long-lasting protection against a wide range of diseases. Continued research and development in this field hold promise for revolutionizing the way we approach vaccination and disease prevention in the future.

So, the integration of nanoparticles into mRNA vaccine formulations represents a paradigm shift in vaccine design and delivery. This review provides a comprehensive analysis of the diverse roles of nanoparticles in enhancing the stability, efficacy, and immunogenicity of mRNA vaccines. By elucidating these advancements, this review aims to highlight the transformative potential of nanoparticles in advancing vaccine technologies, underscoring their critical role in combating infectious diseases and improving global health outcomes.

## 2. Nanoparticles in Vaccine Delivery

### 2.1. Definition and Types of Nanoparticles

Nanoparticles are tiny particles with dimensions measured in nanometers (1–100 nm); they play a crucial role in the field of nanomedicine, particularly in vaccine delivery. Their small size and large surface area-to-volume ratio allow for unique interactions with biological systems, enabling improved delivery and efficacy of vaccines [18].

#### Types of Nanoparticles in Vaccine Delivery

**Figure 3:**
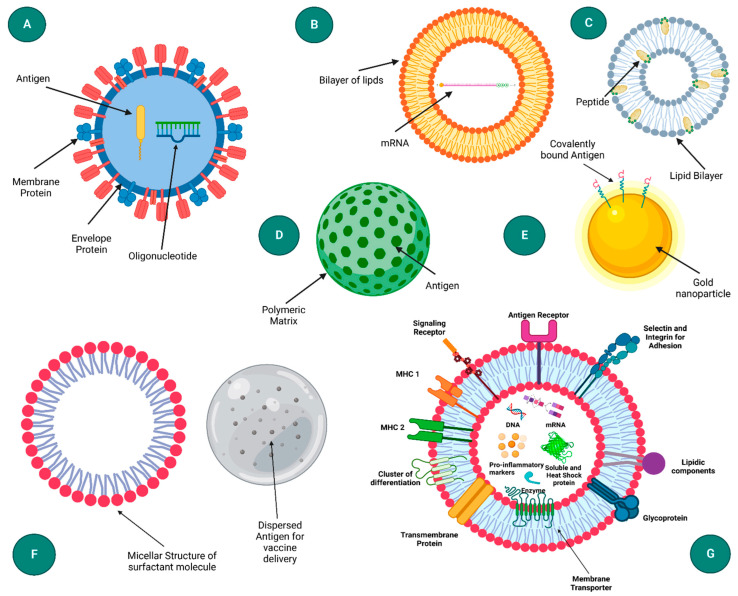
Diagram illustrating various nanoparticle-based delivery systems: (**A**) virus-like particle, (**B**) liposome, (**C**) immune-stimulating complex (ISCOM), (**D**) polymeric nanoparticle, (**E**) inorganic nanoparticle, (**F**) emulsion, and (**G**) exosome. Reprinted with permission from Ref. [18].

1. **Lipid Nanoparticles (LNPs)**: LNPs are the most widely used nanoparticle system for delivering mRNA vaccines. They consist of ionizable lipids, cholesterol, phospholipids, and polyethylene glycol (PEG)-lipids, which form a protective vesicle around mRNA. LNPs enhance the stability of mRNA and facilitate its delivery into the cytoplasm of target cells. The success of Pfizer-BioNTech’s BNT162b2 and Moderna’s mRNA-1273 vaccines against COVID-19 underscores the efficacy of LNPs in mRNA delivery [19].
2. **Polymer-Based Nanoparticles**: Polymers such as poly(lactic-co-glycolic acid) (PLGA) and chitosan are used to create nanoparticles that encapsulate mRNA, protecting it from degradation and enhancing its delivery. These biodegradable and biocompatible polymers offer a sustained release of the mRNA and can be engineered to target specific tissues [20].
3. **Inorganic Nanoparticles**: Inorganic materials like gold, silica, and iron oxide are used to create nanoparticles that can deliver mRNA. Gold nanoparticles, for instance, can be functionalized with various biomolecules to improve targeting and uptake by cells. These nanoparticles also have unique optical and magnetic properties that can be exploited for imaging and diagnostic purposes [21].
4. **Hybrid Nanoparticles**: Combining organic and inorganic materials, hybrid nanoparticles leverage the benefits of both types. For example, a lipid-coated silica nanoparticle can combine the stability and functionalization capabilities of inorganic materials with the biocompatibility and delivery efficiency of lipid nanoparticles [22].
5. **Dendrimers**: These are highly branched, star-shaped macromolecules with a well-defined, monodisperse structure. Dendrimers can be used to encapsulate mRNA and protect it from enzymatic degradation, and their surface can be modified to improve cellular uptake and targeting [23].
6. **Virus-Like Particles (VLPs)**: VLPs mimic the structure of viruses but are non-infectious as they lack viral genetic material. Figure 3 presents a schematic representation of diverse nanoparticle-based delivery systems, each with distinct structural characteristics and applications. The virus-like particle (A) mimics the structure of natural viruses, enhancing cellular uptake and immune response. Liposomes (B) are spherical vesicles with a lipid bilayer, commonly used for drug and gene delivery due to their biocompatibility. Immune-stimulating complexes (C) are cage-like structures that can efficiently deliver antigens to immune cells, promoting strong immune responses. Polymeric nanoparticles (D) offer tunable properties for sustained release, while inorganic nanoparticles (E) provide unique optical and magnetic properties for diagnostics and therapy. Emulsions (F) are mixtures of immiscible liquids used for the delivery of hydrophobic drugs. Lastly, exosomes (G) are cell-derived vesicles that naturally facilitate intercellular communication and are being explored for targeted delivery applications [18]. They can be engineered to carry mRNA and are particularly effective in inducing strong immune responses due to their resemblance to natural pathogens [24].

### 2.2. Advantages of Nanoparticles in Vaccine Delivery

The use of nanoparticles in vaccine delivery offers numerous advantages that enhance the efficacy, stability, and safety of vaccines. These advantages have been demonstrated across various studies and are critical in the development of next-generation vaccines.

#### 2.2.1. Enhanced Stability and Protection

Nanoparticles protect mRNA from enzymatic degradation and hydrolysis, which are significant challenges due to the inherent instability of mRNA. By encapsulating mRNA within nanoparticles, it is shielded from nucleases in the extracellular environment, thus maintaining its integrity until it reaches the target cells [25]. For instance, lipid nanoparticles (LNPs) have been shown to stabilize mRNA effectively, ensuring it remains intact and functional during delivery [26].

#### 2.2.2. Improved Delivery and Uptake

Nanoparticles facilitate the efficient delivery of mRNA into cells. Their small size and surface properties allow them to navigate biological barriers and be taken up by cells through endocytosis. Once inside the cell, nanoparticles can release mRNA into the cytoplasm, where it is translated into the desired protein antigen [27]. LNPs, for example, are designed to fuse with the endosomal membrane, releasing mRNA into the cytoplasm and thereby enhancing delivery efficiency [28].

#### 2.2.3. Targeted Delivery

Nanoparticles can be engineered to target specific cells or tissues, enhancing the vaccine’s efficacy and reducing potential side effects. This targeting can be achieved by functionalizing the surface of nanoparticles with ligands, antibodies, or peptides that bind specifically to receptors on the target cells [29]. For example, nanoparticles targeting dendritic cells, which are key antigen-presenting cells, can significantly enhance the immune response by ensuring that the mRNA is delivered directly to cells involved in initiating immune responses [30].

#### 2.2.4. Enhanced Immune Response

Nanoparticles can enhance the immune response to the delivered mRNA by acting as adjuvants, which are substances that boost the body’s immune response to an antigen. Some nanoparticles, such as certain lipid-based and polymer-based particles, can inherently possess adjuvant properties or can be co-formulated with adjuvants to further enhance immunogenicity [31]. For example, cationic lipid nanoparticles can activate immune cells and promote the production of pro-inflammatory cytokines, thereby enhancing the overall immune response to the mRNA-encoded antigen [32].

#### 2.2.5. Versatility and Scalability

Nanoparticles offer a versatile platform that can be adapted for different types of mRNA vaccines, targeting various diseases. The same nanoparticle delivery system can be used to deliver different mRNA sequences by simply changing the encoded antigen, making it a highly adaptable technology for rapid vaccine development [33]. Additionally, nanoparticle-based vaccine formulations can be manufactured at scale using standardized processes, ensuring consistency and efficiency in production [34].

#### 2.2.6. Reduced Dosage and Side Effects

By improving the delivery and stability of mRNA, nanoparticles can enable lower vaccine doses to achieve the desired immune response, which can reduce the risk of side effects associated with higher doses [35]. This is particularly important for vaccines administered to individuals with compromised immune systems or for repeated dosing, where minimizing adverse reactions is crucial.

#### 2.2.7. Broad Applicability

The principles of nanoparticle delivery systems are broadly applicable across different types of vaccines beyond mRNA, including DNA vaccines, protein subunit vaccines, and viral vector vaccines. This broad applicability enhances the potential impact of nanoparticle-based delivery systems in improving vaccine design and development across various platforms [36]. Ultimately, the utilization of nanoparticles in vaccine delivery represents a significant advancement in the field of vaccinology. By enhancing stability, delivery efficiency, and immunogenicity, nanoparticles address many of the challenges associated with traditional vaccine platforms and open new avenues for the development of safe, effective, and versatile vaccines. The ongoing research and development of nanoparticle-based vaccine delivery systems hold great promise for improving global health outcomes by enabling rapid and targeted responses to emerging infectious diseases and other health threats.

## 3. Mechanisms of Nanoparticle-Enhanced mRNA Vaccines

### 3.1. Improved Delivery and Uptake

Nanoparticle systems have significantly advanced the delivery and uptake of mRNA vaccines, addressing several challenges associated with conventional delivery methods. The improved delivery and uptake mechanisms primarily stem from the unique physicochemical properties of nanoparticles, such as their size, shape, surface charge, and functionalization capabilities.

#### 3.1.1. Size and Shape

The small size of nanoparticles (1–100 nm) facilitates their penetration through biological barriers and uptake by cells. This nanoscale size allows nanoparticles to navigate through the extracellular matrix and enter cells via endocytosis, a process wherein the cell membrane engulfs the nanoparticles, forming an endosome [37]. Studies have shown that nanoparticles within the size range of 20–200 nm are optimal for cellular uptake, with smaller nanoparticles (around 50 nm) demonstrating superior efficiency due to their ability to evade phagocytosis by macrophages and other immune cells. For instance, Kinnear et al. and Hoshyar et al. demonstrated in two different experiments that spherical gold nanoparticles of approximately 50 nm in size were internalized more efficiently by mammalian cells compared to both smaller and larger particles [37,38].

The shape of nanoparticles also influences their cellular uptake. Spherical nanoparticles are generally preferred for uniform distribution and efficient internalization. However, rod-shaped and other anisotropic nanoparticles can exhibit different internalization pathways and cellular interactions, which might be advantageous for specific applications. The spherical nanoparticles were primarily internalized via clathrin-mediated endocytosis, rod-shaped nanoparticles entered cells through alternative pathways, including caveolae-mediated endocytosis and macropinocytosis. This suggests that the shape of nanoparticles can be strategically engineered to target specific cellular uptake mechanisms, enhancing their utility in various biomedical applications. The shape of nanoparticles also influences their cellular uptake. Spherical nanoparticles are generally preferred for uniform distribution and efficient internalization. However, rod-shaped and other anisotropic nanoparticles can exhibit different internalization pathways and cellular interactions, which might be advantageous for specific applications [39].

#### 3.1.2. Surface Charge

The surface charge of nanoparticles plays a crucial role in their interaction with cell membranes. Positively charged (cationic) nanoparticles are more readily taken up by cells due to electrostatic interactions with the negatively charged cell membranes. This enhanced interaction facilitates the binding and subsequent endocytosis of the nanoparticles. However, excessive positive charge can lead to cytotoxicity, so a balance must be maintained to ensure both efficient delivery and biocompatibility [40].

#### 3.1.3. Functionalization

The functionalization of nanoparticle surfaces with targeting ligands, such as antibodies, peptides, or aptamers, enhances the specificity and efficiency of delivery. These ligands bind to specific receptors on the target cells, facilitating receptor-mediated endocytosis. This targeted approach not only improves the uptake of nanoparticles by the desired cells but also minimizes off-target effects and potential toxicity [41].

For instance, lipid nanoparticles (LNPs), widely used in mRNA vaccine delivery, are functionalized with polyethylene glycol (PEG) to enhance their stability and circulation time in the bloodstream. PEGylation reduces opsonization and clearance by the mononuclear phagocyte system, thereby improving the bioavailability of the mRNA cargo [42].

### 3.2. Enhanced Immune Response

Nanoparticles not only improve the delivery and uptake of mRNA vaccines but also play a crucial role in enhancing the immune response. The immunogenicity of mRNA vaccines can be significantly amplified through the use of nanoparticles by leveraging their intrinsic adjuvant properties and the ability to target specific immune cells.

#### 3.2.1. Endosomal Escape

Once inside the cell, nanoparticles must escape the endosome to release their mRNA cargo into the cytoplasm. This step is critical because mRNA needs to reach the cytoplasm to be translated into the target protein. Various strategies have been employed to facilitate endosomal escape, including the use of pH-sensitive lipids that destabilize the endosomal membrane at an acidic pH, fusogenic peptides that mimic viral fusion proteins, and proton sponge effect-inducing polymers that cause osmotic swelling and rupture of the endosome [43].

LNPs, for example, are designed with ionizable lipids that become protonated in the acidic environment of the endosome, leading to membrane fusion and the release of mRNA into the cytoplasm. This mechanism significantly enhances the efficiency of mRNA delivery and subsequent protein expression [44].

**Figure 4:**
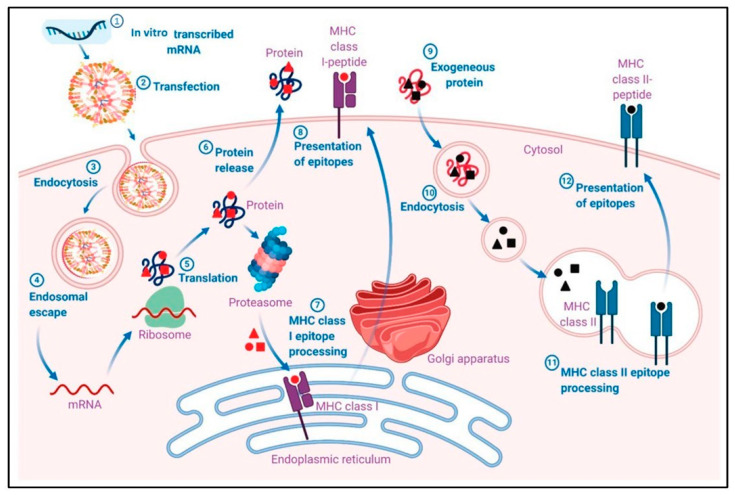
Pharmacological mechanism of adaptive immune responses induced by mRNA-LNP vaccines. (1) In vitro transcribed mRNA is encapsulated into a lipid nanoparticle (LNP). (2) Transfection of mRNA-LNP vaccine molecules into the host cells, using specialized lipids on the surface of the LNPs. (3) Endocytosis of mRNA-LNP. (4) Endosomal escape of mRNA to the cytosol after endocytosis-mediated internalization. (5) Intracellular translation of mRNA by the host cell ribosomes into the desired antigen protein. (6) The antigenic protein is released outside the cell, or the antigenic protein is degraded by a proteosome, exposing the antigenic sites. (7) Major histocompatibility complex I (MHC I) epitope presentation of the MHC I to the cell membrane for antigen presentation (APC). MHC I presents the epitope to CD8+ T cells. (9) The exogenous protein released earlier can become degraded and presented via MHC II epitopes. The extracellular antigen can be recognized by B cells, leading to B cell maturation. Reprinted with permission from Ref. [45].

The use of nanoparticles in mRNA vaccine delivery enhances cellular uptake, through their optimized size, shape, surface charge, and functionalization, and facilitates endosomal escape, ensuring that mRNA reaches the cytoplasm where it can be translated into the desired antigenic protein. Figure 4 illustrates the pharmacological mechanism through which mRNA-LNP vaccines induce adaptive immune responses. Initially, mRNA is encapsulated into lipid nanoparticles (LNPs) and delivered into host cells via transfection. Once inside the cell, mRNA escapes the endosome and is translated into the target antigen by the host’s ribosomes. The produced antigen can be processed and presented on the cell surface by major histocompatibility complex I (MHC I) molecules, which then activate CD8+ T cells. Additionally, exogenously released antigens can be processed and presented by MHC II molecules, leading to the activation of B cells and the production of antibodies. This dual pathway ensures a robust and comprehensive immune response. [45].

#### 3.2.2. Adjuvant Properties

Nanoparticles can act as adjuvants themselves or be co-formulated with adjuvants to boost immune response. Certain nanoparticles, such as cationic lipids, can activate innate immune pathways, leading to the production of cytokines and chemokines that enhance the adaptive immune response. For example, the lipid nanoparticles (LNPs) used in mRNA vaccines can trigger Toll-like receptor (TLR) signaling, which plays a vital role in the activation of the innate immune system [46].

Additionally, nanoparticles can be designed to deliver mRNA alongside traditional adjuvants, such as aluminum salts, saponins, or synthetic TLR agonists. These combinations can synergistically enhance the immune response by activating multiple pathways, resulting in a more robust and durable immunity [47].

#### 3.2.3. Targeting Dendritic Cells

Dendritic cells (DCs) are crucial antigen-presenting cells that initiate and modulate the adaptive immune response. Targeting nanoparticles to DCs can significantly enhance the efficacy of mRNA vaccines. Nanoparticles can be functionalized with ligands that specifically bind to receptors on DCs, such as DEC-205, DC-SIGN, or mannose receptors, facilitating the uptake and presentation of the mRNA-encoded antigen [48]. For example, gold nanoparticles functionalized with mannose residues have been shown to target DCs effectively, leading to the enhanced uptake and presentation of the antigen and, subsequently, stronger T cell responses [49].

#### 3.2.4. Inducing Cytotoxic T Lymphocyte Responses

Effective mRNA vaccines need to induce strong cytotoxic T lymphocyte (CTL) responses, especially for infections and cancers where killing infected or malignant cells is essential. Nanoparticles can enhance CTL responses by ensuring the efficient delivery of mRNA to the cytoplasm, where it can be processed and presented on MHC class I molecules to CD8+ T cells. This process is critical for the activation and expansion of CTLs that can target and destroy infected or cancerous cells [50].

LNPs, for example, have been shown to induce potent CTL responses in preclinical models, translating into effective antiviral and antitumor immunity [51].

#### 3.2.5. Promoting Humoral Immunity

In addition to cellular immunity, nanoparticles can enhance humoral immunity by facilitating the delivery of mRNA to B cells and follicular DCs, leading to the production of high-affinity antibodies. The presentation of the antigen in a native conformation is crucial for B cell activation and affinity maturation. Nanoparticles can stabilize and present the mRNA-encoded protein in a manner that mimics the natural pathogen, thus improving B cell recognition and antibody production [52].

#### 3.2.6. Multivalent Display of Antigens

Nanoparticles can be engineered to display multiple copies of the antigen on their surface, mimicking the repetitive patterns found on pathogens. This multivalent display can enhance the cross-linking of B cell receptors, leading to stronger B cell activation and antibody responses. For example, virus-like particles (VLPs) presenting multiple copies of the antigen have been shown to induce robust antibody responses due to their high density and repetitive structure [53].

In conclusion, nanoparticles enhance the immune response to mRNA vaccines by acting as adjuvants, targeting dendritic cells, promoting CTL and humoral responses, and enabling the multivalent display of antigens. These mechanisms collectively contribute to the generation of robust and long-lasting immunity, making nanoparticles a pivotal component in the development of effective mRNA vaccines.

### 3.3. Protection and Stability of mRNA

The stability of mRNA is a critical factor in the development and efficacy of mRNA vaccines. Nanoparticles play a vital role in protecting mRNA from degradation and ensuring its stability throughout the delivery process. This section discusses the various strategies employed by nanoparticles to enhance the protection and stability of mRNA.

#### 3.3.1. Protection from RNases

One of the primary challenges in mRNA vaccine development is the susceptibility of mRNA to degradation by ribonucleases (RNases), which are abundant in biological fluids. Nanoparticles encapsulate mRNA, shielding it from RNase activity and preventing degradation. For instance, lipid nanoparticles (LNPs) form a protective lipid bilayer around mRNA, effectively isolating it from extracellular RNases and enhancing its stability [54].

#### 3.3.2. Chemical Modification of mRNA

Chemical modifications of mRNA, such as the incorporation of modified nucleotides (e.g., pseudouridine or 5-methylcytidine), can enhance its stability and translation efficiency. These modifications reduce the recognition of mRNA by the innate immune system, decreasing its degradation and increasing protein production. Nanoparticles can be designed to deliver chemically modified mRNA, further improving its stability and immunogenicity [55].

#### 3.3.3. Encapsulation Techniques

Encapsulation techniques used in nanoparticle formulation are crucial for protecting mRNA. Techniques such as microfluidic mixing allow for the precise encapsulation of mRNA within nanoparticles, ensuring uniform particle size and high encapsulation efficiency. These methods not only protect mRNA but also enhance its stability by preventing aggregation and ensuring controlled release [56].

#### 3.3.4. Lyophilization and Storage

Nanoparticles can be lyophilized (freeze-dried) to improve the long-term stability of mRNA vaccines. Lyophilization removes water from the nanoparticle formulation, preventing the hydrolytic degradation of mRNA and extending the shelf life of the vaccine. This process is particularly important for the storage and distribution of mRNA vaccines, as it allows them to be stored at higher temperatures compared to liquid formulations [57].

#### 3.3.5. Protection against Mechanical and Thermal Stress

Nanoparticles provide mechanical stability to mRNA, protecting it from shear forces and thermal stress during manufacturing, storage, and administration. The robust structure of nanoparticles ensures that mRNA remains intact and functional throughout the vaccine’s lifecycle. For example, polymer-based nanoparticles, such as those made from poly(lactic-co-glycolic acid) (PLGA), offer excellent protection against mechanical stress, enhancing the stability and efficacy of the mRNA payload [58].

#### 3.3.6. Improving Intracellular Stability

Once inside the cell, mRNA must remain stable long enough to be translated into the target protein. Nanoparticles enhance intracellular stability by facilitating the rapid release of mRNA into the cytoplasm, where it is less susceptible to degradation compared to the extracellular environment. Ionizable lipids in LNPs, for example, undergo protonation in the acidic endosomal environment, triggering endosomal escape and release of mRNA into the cytoplasm [59].

In conclusion, nanoparticles significantly enhance the protection and stability of mRNA by shielding it from RNase degradation, incorporating chemical modifications, employing efficient encapsulation techniques, facilitating lyophilization, and providing mechanical and thermal stability. These strategies collectively ensure that mRNA remains stable and functional throughout the vaccine development and delivery process, ultimately improving the efficacy of mRNA vaccines.

## 4. Types of Nanoparticles Used in mRNA Vaccines

### 4.1. Lipid Nanoparticles

Lipid nanoparticles (LNPs) are among the most widely used delivery systems for mRNA vaccines. Their design and composition are pivotal in determining the efficacy and stability of the mRNA they carry.

#### 4.1.1. Composition and Structure

LNPs typically consist of ionizable lipids, phospholipids, cholesterol, and polyethylene glycol (PEG)-lipid conjugates. The ionizable lipids are crucial because they remain neutral at a physiological pH but become positively charged in the acidic endosomal environment, facilitating the endosomal escape of mRNA [60]. Phospholipids contribute to the formation of the lipid bilayer, while cholesterol provides structural integrity and stability. PEG-lipid conjugates extend the half-life of LNPs by reducing protein adsorption and opsonization.

#### 4.1.2. Mechanism of Action

The mechanism by which LNPs deliver mRNA involves several steps: encapsulation, systemic delivery, cellular uptake, endosomal escape, and mRNA release into the cytoplasm. Upon intramuscular injection, LNPs protect mRNA from degradation by RNases in the extracellular environment. The nanoparticles are then taken up by cells via endocytosis. Once inside the endosome, the ionizable lipids become protonated, leading to the destabilization of the endosomal membrane and release of mRNA into the cytoplasm where it can be translated into protein [61].

#### 4.1.3. Clinical Success

LNPs have shown remarkable success in clinical settings, particularly with the development of COVID-19 mRNA vaccines such as those by Pfizer-BioNTech (BNT162b2) and Moderna (mRNA-1273). These vaccines demonstrated high efficacy and safety profiles, largely attributed to the advanced LNP delivery systems that ensured efficient mRNA delivery and robust immune responses [14,19].

#### 4.1.4. Advantages

LNPs offer several advantages including high encapsulation efficiency, scalability for mass production, and the ability to incorporate various modifications to enhance stability and targeting. The use of PEGylation significantly improves the pharmacokinetics and biodistribution of LNPs, ensuring prolonged circulation time and enhanced delivery to target tissues [62].

#### 4.1.5. Challenges and Future Directions

Despite their success, LNPs face challenges such as the potential immunogenicity of the PEG component, which can lead to anti-PEG antibodies and reduced efficacy upon repeated dosing [63]. Future research is focused on developing alternative stealth coatings and optimizing lipid compositions to overcome these limitations and enhance the safety and efficacy of LNP-based mRNA vaccines.

### 4.2. Polymer-Based Nanoparticles

Polymer-based nanoparticles offer another versatile platform for mRNA delivery. These nanoparticles can be engineered from a variety of natural and synthetic polymers, each with distinct properties that can be tailored for specific applications.

#### 4.2.1. Types of Polymers

Common polymers used in mRNA delivery include poly(lactic-co-glycolic acid) (PLGA), polyethylenimine (PEI), chitosan, and poly(β-amino esters) (PBAEs). PLGA is widely used due to its biocompatibility and biodegradability. It can protect mRNA from enzymatic degradation and provide sustained release profiles. PEI is known for its high transfection efficiency, but its use is limited by the cytotoxicity associated with high-molecular-weight PEI. Chitosan, a natural polysaccharide, offers biocompatibility and mucoadhesive properties, making it suitable for mucosal delivery. PBAEs provide a balance between efficiency and biocompatibility due to their biodegradable and tunable nature [64].

#### 4.2.2. Mechanism of Action

Polymer-based nanoparticles encapsulate mRNA through various methods, including emulsion, nanoprecipitation, and ionic gelation. These nanoparticles deliver mRNA into cells via endocytosis, similar to LNPs. The release of mRNA into the cytoplasm often relies on the polymer’s ability to disrupt endosomal membranes, a property enhanced by incorporating pH-sensitive or proton-sponge effects [65].

#### 4.2.3. Advantages

Polymer-based nanoparticles offer high versatility in design and functionality. They can be engineered to control the release rate of mRNA, enhance stability, and target specific tissues or cells by surface modification with ligands or antibodies. Additionally, polymers such as PLGA have a well-established safety profile, facilitating regulatory approval [66].

#### 4.2.4. Challenges and Future Directions

The main challenges include potential cytotoxicity, especially with cationic polymers like PEI, and the need for precise control over polymer degradation rates to ensure the timely release of mRNA. Future research is focused on developing new polymers and hybrid systems that combine the advantages of different materials to enhance delivery efficiency and reduce side effects [67,68].

### 4.3. Inorganic Nanoparticles

Inorganic nanoparticles, such as gold nanoparticles (AuNPs), silica nanoparticles, and quantum dots, provide unique advantages for mRNA delivery due to their distinct physicochemical properties.

#### 4.3.1. Types of Inorganic Nanoparticles

Gold nanoparticles (AuNPs) are particularly attractive due to their biocompatibility, ease of functionalization, and ability to induce localized hyperthermia for cancer therapy. Silica nanoparticles offer high surface area and porosity, which can be utilized for loading large amounts of mRNA. Quantum dots, composed of semiconductor materials, provide fluorescence properties useful for tracking and imaging delivery processes [69,70,71,72].

#### 4.3.2. Mechanism of Action

Inorganic nanoparticles deliver mRNA through various mechanisms, often involving surface modifications with cationic polymers or lipids to facilitate mRNA binding and cellular uptake. The high surface area and functionalizable surface of these nanoparticles allow for efficient mRNA encapsulation and targeted delivery. For instance, AuNPs can be functionalized with thiol-modified mRNA for stable conjugation and efficient delivery [73].

#### 4.3.3. Advantages

Inorganic nanoparticles offer robustness and stability, making them suitable for various biomedical applications. Their unique optical and electronic properties enable real-time tracking and imaging, providing valuable insights into the delivery process. Moreover, the ease of functionalization allows for the development of multifunctional delivery systems that can combine therapeutic and diagnostic functions (theranostics) [74].

#### 4.3.4. Challenges and Future Directions

The main challenges include potential toxicity and long-term persistence in the body. Inorganic nanoparticles often require thorough biocompatibility and biosafety evaluations. Future research aims to address these issues by developing biodegradable inorganic nanoparticles and hybrid systems that combine the stability of inorganic materials with the biocompatibility of organic components [75].

### 4.4. Hybrid Nanoparticles

Hybrid nanoparticles, which combine the properties of different materials, offer a promising approach for mRNA delivery. These systems aim to leverage the advantages of each component while mitigating their individual limitations.

#### 4.4.1. Types of Hybrid Nanoparticles

Hybrid nanoparticles can be composed of combinations such as lipid–polymer, lipid–inorganic, and polymer–inorganic materials. For example, lipid–polymer hybrid nanoparticles (LPHNPs) combine the biocompatibility and membrane fusion capabilities of lipids with the structural integrity and controlled release properties of polymers. Similarly, lipid–inorganic hybrids can combine the stability of inorganic nanoparticles with the flexibility and functionalization potential of lipids [76].

#### 4.4.2. Mechanism of Action

Hybrid nanoparticles deliver mRNA through complex mechanisms involving multiple stages of interaction with biological systems. The hybrid nature allows for the incorporation of various functionalities, such as targeted delivery, controlled release, and imaging capabilities. For instance, lipid–polymer hybrids can encapsulate mRNA within the polymer core while the lipid shell facilitates cellular uptake and endosomal escape [77].

#### 4.4.3. Advantages

The primary advantage of hybrid nanoparticles is their ability to integrate the beneficial properties of different materials. They offer enhanced stability, controlled release, and targeted delivery while maintaining biocompatibility. Hybrid systems can also be designed to provide multifunctional capabilities, such as combining therapeutic delivery with diagnostic imaging (theranostics) [78].

#### 4.4.4. Challenges and Future Directions

The complexity of hybrid nanoparticle design and manufacturing poses challenges for reproducibility and scalability. Ensuring consistent quality and performance requires advanced fabrication techniques and thorough characterization. Future research will be focused on optimizing the synthesis and functionalization of hybrid nanoparticles to enhance their efficacy and safety for mRNA delivery [79]. The use of various types of nanoparticles, including lipid, polymer-based, inorganic, and hybrid nanoparticles, represents a significant advancement in the delivery of mRNA vaccines. Each type offers unique advantages and faces specific challenges, and their continued development promises to enhance the efficacy, stability, and safety of mRNA-based therapeutics.

## 5. Synthesis and Characterization of Nanoparticles Used in mRNA Vaccines

### 5.1. Methods of Synthesis

The synthesis of nanoparticles for mRNA vaccine delivery involves various methods aimed at achieving controlled size, shape, surface properties, and biocompatibility. These nanoparticles serve as carriers to protect mRNA from degradation, facilitate cellular uptake, and enhance vaccine efficacy. Here, we explore several prominent methods along with their applications and implications in mRNA vaccine development.

**Polymeric Nanoparticles:** Polymeric nanoparticles are widely investigated due to their versatility, biocompatibility, and ability to encapsulate and deliver mRNA effectively. Methods such as nanoprecipitation, emulsion solvent evaporation, and electrospinning are commonly employed for their synthesis. For instance, poly(lactic-co-glycolic acid) (PLGA) nanoparticles synthesized via nanoprecipitation have shown promise in encapsulating mRNA, protecting it from enzymatic degradation, and facilitating controlled release kinetics [80].

**Lipid-Based Nanoparticles:** Lipid nanoparticles (LNPs) are another pivotal class of nanoparticles for mRNA vaccine delivery. LNPs typically consist of ionizable lipids, cholesterol, and PEGylated lipids, which stabilize and protect mRNA during delivery. Methods such as microfluidics and double emulsion techniques are utilized to produce LNPs with precise particle sizes and surface characteristics. LNPs have demonstrated high transfection efficiency and immune response induction, making them a leading candidate for mRNA vaccine formulations [33].

**Inorganic Nanoparticles:** Inorganic nanoparticles, including gold nanoparticles, silica nanoparticles, and quantum dots, offer unique physical and chemical properties that can be exploited in mRNA vaccine design. Synthesis methods involve chemical reduction, sol–gel processes, and seed-mediated growth. These nanoparticles can serve as adjuvants, carriers, or imaging agents in vaccine formulations, enhancing stability and targeting specific immune responses [18].

**Hybrid Nanoparticles:** Hybrid nanoparticles combine the advantages of different materials to achieve synergistic effects in mRNA vaccine delivery. For example, lipid–polymer hybrid nanoparticles merge the biocompatibility of lipids with the structural integrity of polymers, offering enhanced stability and controlled release profiles. These hybrids are designed to overcome the limitations of individual materials and optimize mRNA delivery efficiency [14].

Each synthesis method influences the physicochemical properties of nanoparticles, affecting their stability, biocompatibility, and therapeutic efficacy in mRNA vaccines. The choice of method depends on the desired characteristics of the nanoparticle and the specific requirements of the vaccine formulation [34].

### 5.2. Techniques for Characterization

Characterizing nanoparticles is essential for understanding their structure, stability, and interactions with biological systems, and crucial for optimizing mRNA vaccine formulations. A variety of advanced techniques are employed to assess nanoparticle properties comprehensively.

**Dynamic Light Scattering (DLS):** DLS measures the size distribution and colloidal stability of nanoparticles in solution. It provides information on particle size, aggregation state, and surface charge, which are critical for assessing nanoparticle behavior in physiological environments. DLS is valuable for optimizing formulation parameters and ensuring consistent vaccine efficacy [81].

**Transmission Electron Microscopy (TEM):** TEM offers the high-resolution imaging of nanoparticles, revealing details about their morphology, size, and internal structure. It is indispensable for confirming uniformity and integrity in mRNA-loaded nanoparticles, identifying aggregation or surface modifications that could impact vaccine performance [2].

**Fourier Transform Infrared Spectroscopy (FTIR):** FTIR spectroscopy identifies functional groups and chemical bonds present on nanoparticle surfaces or within polymer matrices. It is used to verify the encapsulation of mRNA and monitor structural changes during synthesis or storage, ensuring the stability and functionality of vaccine formulations [82].

**X-ray Diffraction (XRD):** XRD analyzes the crystalline structure of inorganic nanoparticles, providing insights into their composition, phase purity, and stability. This technique is particularly useful for characterizing nanoparticles used as adjuvants or carriers in mRNA vaccines, helping to optimize their physicochemical properties for enhanced efficacy [14,33].

**Zeta Potential Analysis:** Zeta potential measurement evaluates the surface charge of nanoparticles, influencing their stability, dispersibility, and interactions with biological membranes. For example, a high zeta potential (either positive or negative, typically above ±30 mV) generally indicates strong electrostatic repulsion between particles, preventing aggregation and leading to a more stable dispersion. This is crucial for ensuring efficient cellular uptake and minimizing cytotoxicity in mRNA vaccine delivery systems [83]. Conversely, a zeta potential close to 0 mV suggests that the particles are more likely to aggregate due to insufficient repulsive forces, which is unacceptable as this can lead to the reduced efficacy and increased toxicity of the vaccine.

**Nuclear Magnetic Resonance (NMR) Spectroscopy:** NMR spectroscopy elucidates the molecular dynamics and interactions of nanoparticles with mRNA payloads or targeting ligands. For example, NMR can reveal how the conformation of a nanoparticle changes upon binding to an mRNA strand or a targeting ligand, providing critical insights into the nanoparticle’s structure–function relationship. This information guides researchers in refining nanoparticle designs to enhance stability and delivery efficiency. An unacceptable outcome would be the detection of irregular or unstable binding patterns, indicating that the nanoparticles might not effectively protect the mRNA payload or properly interact with target cells, potentially compromising the efficacy and safety of the mRNA vaccine delivery system [84].

The integration of these characterization techniques enables a comprehensive evaluation of nanoparticle formulations for mRNA vaccines, ensuring robust performance and safety in clinical applications. Each technique contributes specific data essential for optimizing nanoparticle design and enhancing vaccine efficacy.

## 6. Applications and Case Studies of Nanoparticles in mRNA Vaccines

### 6.1. Current mRNA Vaccines Utilizing Nanoparticles

Nanoparticles play a pivotal role in enhancing the efficacy and stability of mRNA vaccines by protecting mRNA molecules, facilitating cellular uptake, and modulating immune responses. Several current mRNA vaccine formulations utilize nanoparticles, each employing distinct nanoparticle types and strategies to optimize vaccine delivery and performance.

**Lipid Nanoparticle (LNP)-Based Vaccines:** Lipid nanoparticles are extensively employed in mRNA vaccine development due to their ability to encapsulate mRNA, protect it from degradation, and facilitate its delivery into target cells. The Pfizer-BioNTech and Moderna COVID-19 vaccines are prominent examples utilizing LNPs to deliver mRNA encoding spike proteins of SARS-CoV-2. These LNPs consist of ionizable lipids, cholesterol, and PEGylated lipids, ensuring stability and enhancing cellular uptake through endocytosis mechanisms [26].

**Polymeric Nanoparticle-Based Vaccines:** Polymeric nanoparticles offer another platform for mRNA vaccine delivery, leveraging biocompatible materials like poly(lactic-co-glycolic acid) (PLGA) or chitosan. These nanoparticles can encapsulate mRNA and facilitate controlled release kinetics, contributing to sustained antigen expression and immune stimulation. Research into polymeric nanoparticles for mRNA vaccines focuses on enhancing stability and fine-tuning delivery properties to optimize immune responses [10].

**Figure 5:**
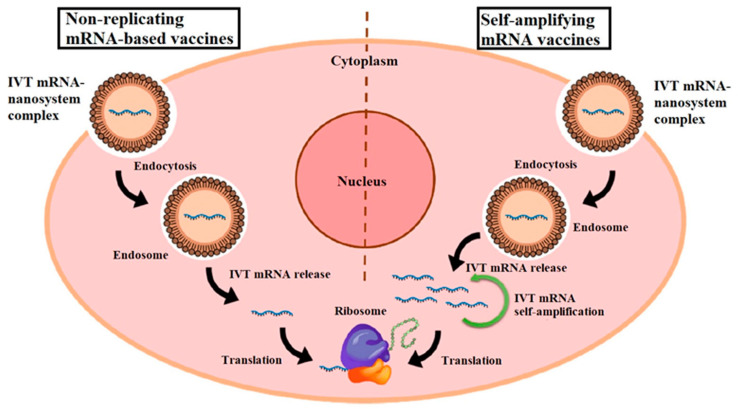
Diagrammatic representation of two types of mRNA vaccines. In vitro transcribed (IVT) mRNA molecules are encapsulated in nanoparticles (NPs), which enter the cell via endocytosis, forming an endosome. Subsequently, the IVT mRNA is released from the complex and binds directly to ribosomes for antigen translation in non-replicating mRNA vaccines. Self-amplifying mRNA (SAM) vaccines, also known as replicons, contain two distinct open reading frames (ORFs). After release from the endosome, one ORF encodes the antigen of interest, while the other encodes proteins necessary for RNA capping and replication. Reprinted with permission from Ref. [85].

**Inorganic Nanoparticle-Based Vaccines:** Inorganic nanoparticles, such as gold nanoparticles or silica nanoparticles, are explored as adjuvants or carriers in mRNA vaccines. Figure 5 illustrates the mechanisms of two types of mRNA vaccines: non-replicating mRNA vaccines and self-amplifying mRNA (SAM) vaccines. In non-replicating mRNA vaccines, in vitro transcribed (IVT) mRNA is delivered into cells via lipid nanoparticles (NPs), which are internalized through endocytosis. mRNA is then released and translated into antigenic proteins directly by ribosomes. Conversely, SAM vaccines, or replicons, contain two open reading frames (ORFs) within mRNA. One ORF encodes the target antigen, while the other ORF encodes proteins essential for RNA replication and capping, amplifying the mRNA’s presence and potentially enhancing the immune response. This diagram highlights the differences in their operational mechanisms and their respective roles in vaccine efficacy [85]. These nanoparticles can be functionalized to enhance antigen presentation, promote immune cell activation, or facilitate targeted delivery. Although less commonly used in current mRNA vaccines compared to LNPs, inorganic nanoparticles hold promise for future vaccine formulations requiring specific immunomodulatory effects [86].

The integration of nanoparticles into mRNA vaccine formulations not only enhances vaccine stability and delivery efficiency but also enables rapid development and scalability during global health emergencies, as demonstrated by the COVID-19 pandemic. Ongoing research continues to refine nanoparticle-based mRNA vaccines to address emerging infectious diseases and other therapeutic applications.

### 6.2. Case Studies and Clinical Trials

Case studies and clinical trials provide critical insights into the efficacy, safety, and translational potential of nanoparticle-based mRNA vaccines in real-world settings. These studies evaluate vaccine performance, immune responses, and patient outcomes, highlighting successes, challenges, and future directions for nanoparticle technology in mRNA vaccine development.

**COVID-19 mRNA Vaccines:** The rapid development and deployment of COVID-19 mRNA vaccines, Pfizer-BioNTech’s BNT162b2 and Moderna’s mRNA-1273, represent landmark achievements in nanoparticle-based vaccine technology. Clinical trials demonstrated high efficacy rates in preventing COVID-19 infection and severe disease, validating the use of LNPs for mRNA delivery on a global scale [60,87].

**Influenza mRNA Vaccines:** Clinical trials investigating influenza mRNA vaccines encapsulated in LNPs have shown promising results in eliciting protective immune responses against seasonal influenza strains. These studies assess the vaccine safety, immunogenicity, and durability of immune responses, paving the way for next-generation influenza vaccines with improved efficacy and broader strain coverage [88].

**Cancer Immunotherapy:** Nanoparticle-based mRNA vaccines are also being explored in cancer immunotherapy, aiming to induce tumor-specific immune responses and enhance antitumor immunity. Clinical trials focus on assessing vaccine safety, antigen specificity, and therapeutic efficacy in various cancer types, highlighting the potential of mRNA-nanoparticle vaccines as personalized treatment options [89].

Case studies and clinical trials underscore the versatility and adaptability of nanoparticle-based mRNA vaccines across diverse disease targets and therapeutic applications. These studies provide crucial data for regulatory approvals, vaccine deployment strategies, and future advancements in mRNA vaccine technology.

## 7. Challenges and Future Prospects of Nanoparticle-Enhanced mRNA Vaccines

### 7.1. Potential Risks and Limitations

While nanoparticle-enhanced mRNA vaccines offer promising advantages in vaccine delivery and efficacy, several challenges and potential risks need to be addressed to ensure their safety and efficacy in clinical applications.

**Immune Responses and Safety Concerns:** One of the primary concerns with nanoparticle-based mRNA vaccines is the potential for inducing unintended immune responses or adverse reactions. Nanoparticles can trigger immune activation through various mechanisms, including complement activation, cytokine release, or immune cell recognition. These immune responses may lead to local inflammation, systemic reactions, or long-term immunogenicity issues, necessitating thorough preclinical and clinical evaluation [90].

**Biocompatibility and Toxicity:** The biocompatibility of nanoparticles is crucial for their safe application in mRNA vaccines. Some nanoparticle materials, especially inorganic nanoparticles, may exhibit toxicity due to their physicochemical properties or degradation products. Assessing nanoparticle biocompatibility involves comprehensive studies to evaluate potential cytotoxicity, genotoxicity, and organ-specific toxicity profiles, ensuring minimal adverse effects on vaccine recipients [91].

**Manufacturing and Scale-up Challenges:** Achieving consistent nanoparticle synthesis at large scales while maintaining batch-to-batch reproducibility is another significant challenge. Variability in nanoparticle size, composition, or surface properties can impact vaccine stability, efficacy, and regulatory approval processes. Developing robust manufacturing processes and quality control measures is essential for ensuring the reliability and scalability of nanoparticle-enhanced mRNA vaccines [92].

**Storage and Stability Issues:** Nanoparticle-based mRNA vaccines often require specific storage conditions to maintain their integrity and efficacy. Challenges such as nanoparticle aggregation, mRNA degradation, or loss of vaccine potency during storage and transportation need to be addressed. Innovations in vaccine formulation and packaging strategies are necessary to enhance stability and extend shelf-life, especially in resource-limited settings or during global distribution efforts [93].

**Regulatory and Ethical Considerations:** Navigating regulatory pathways for nanoparticle-based mRNA vaccines involves addressing novel safety concerns and demonstrating comparability with conventional vaccine standards. Ethical considerations include informed consent, risk–benefit assessments, and equitable access to advanced vaccine technologies across diverse populations. Collaborative efforts between regulatory agencies, researchers, and stakeholders are essential to establish transparent guidelines and ensure public confidence in nanoparticle-enhanced mRNA vaccines [94].

### 7.2. Future Directions in Nanoparticle-Enhanced mRNA Vaccines

**Figure 6:**
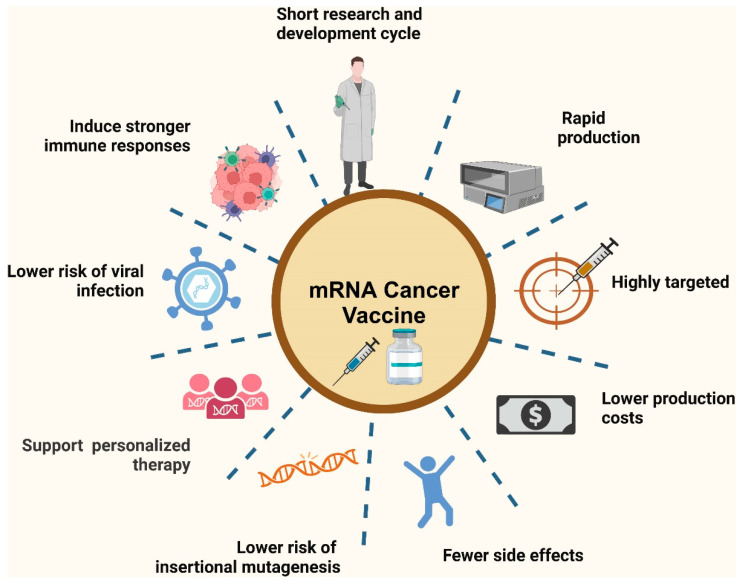
Advantages of mRNA vaccines in cancer therapy. Reprinted with permission from Ref. [89].

Despite the current challenges, nanoparticle-enhanced mRNA vaccines hold significant promise for advancing vaccine technology and addressing global health challenges. Future directions focus on overcoming existing limitations and exploring innovative strategies to maximize vaccine efficacy, safety, and accessibility. Figure 6 showcases the advantages of mRNA vaccines in cancer therapy. These vaccines leverage the ability of mRNA to instruct cells to produce tumor-specific antigens, which can stimulate a targeted immune response against cancer cells. This figure likely highlights key benefits such as the high adaptability of mRNA vaccines for different tumor types, their potential for personalized treatment by encoding unique tumor antigens, and their rapid development capabilities compared to traditional cancer vaccines. Additionally, mRNA vaccines can enhance the specificity and efficacy of immune responses, providing a promising approach for improving cancer treatment outcomes [89].

**Advanced Nanoparticle Design and Optimization:** Continued research aims to optimize nanoparticle design by fine-tuning material composition, surface modifications, and structural properties. Advancements in nanotechnology enable the development of multifunctional nanoparticles capable of targeted delivery, controlled release kinetics, and enhanced immune activation. Integrating smart nanomaterials and biocompatible polymers expands the repertoire of nanoparticle-based vaccine platforms tailored for specific pathogens or therapeutic targets [18].

**Enhanced Delivery Systems and Adjuvant Strategies:** Innovative delivery systems, such as stimuli-responsive nanoparticles or self-assembling structures, offer potential solutions to enhance mRNA stability and intracellular delivery efficiency. The co-delivery of adjuvants or immune stimulants with mRNA payloads enhances antigen presentation and immune response modulation, promoting durable immunity against infectious diseases or cancer. Combining nanotechnology with immunomodulatory strategies fosters synergistic effects and expands the therapeutic applications of mRNA vaccines [52].

**Personalized Medicine and Therapeutic Applications:** Nanoparticle-based mRNA vaccines pave the way for personalized medicine approaches tailored to individual genetic profiles or disease susceptibilities. Advances in mRNA synthesis, nanoparticle formulation, and delivery technologies enable rapid vaccine development against emerging pathogens or variant strains. Harnessing mRNA’s programmable nature and nanoparticle versatility accelerates the translation of precision medicine concepts into clinical practice, offering targeted therapies and prophylactic solutions for diverse healthcare challenges [95].

**Global Access and Vaccine Equity:** Addressing global health disparities requires equitable access to advanced vaccine technologies, including nanoparticle-enhanced mRNA vaccines. Collaborative efforts in technology transfer, capacity-building, and affordability initiatives facilitate global manufacturing capabilities and distribution networks. Leveraging public–private partnerships and international collaborations strengthens pandemic preparedness and resilience, ensuring timely access to lifesaving vaccines for vulnerable populations worldwide [96].

**Integration of Artificial Intelligence and Digital Technologies:** Artificial intelligence (AI) and machine learning (ML) algorithms accelerate vaccine design, nanoparticle optimization, and the predictive modeling of immune responses. The virtual screening of nanoparticle libraries and bioinformatics tools streamline candidate selection, toxicity profiling, and vaccine formulation strategies. Integrating AI-driven platforms with high-throughput screening methodologies revolutionizes vaccine discovery pipelines, fostering rapid innovation and adaptive responses to evolving public health threats [97].

## 8. Conclusions: Enhancing Vaccine Efficacy and Stability with Nanoparticles in mRNA Vaccines

### 8.1. Summary of Key Points

Nanoparticles have emerged as versatile tools in revolutionizing mRNA vaccine development, aiming to enhance vaccine efficacy, stability, and delivery efficiency. This review synthesizes key insights from the current literature to highlight the pivotal role of nanoparticles in advancing the field of mRNA vaccines.

**Role of Nanoparticles in mRNA Vaccine Formulation:** Nanoparticles, particularly lipid nanoparticles (LNPs) and polymeric nanoparticles, serve as effective carriers for mRNA molecules, protecting them from enzymatic degradation and facilitating their efficient delivery into target cells. LNPs, exemplified by the Pfizer-BioNTech and Moderna COVID-19 vaccines, encapsulate mRNA encoding viral antigens, enabling robust immune responses and high vaccine efficacy.

**Advantages of Nanoparticle-Based Delivery Systems:** The use of nanoparticles enhances vaccine stability by preventing premature mRNA degradation and optimizing its bioavailability. Advanced nanoparticle designs, including surface modifications and the incorporation of stabilizing agents, prolong mRNA’s half-life and facilitate controlled release kinetics, which are crucial for sustained antigen expression and prolonged immune stimulation.

**Characterization and Optimization of Nanoparticles:** Critical to nanoparticle-enhanced mRNA vaccines is the characterization of nanoparticle properties using techniques such as dynamic light scattering, transmission electron microscopy, and spectroscopic methods. These analyses ensure uniform particle size distribution, structural integrity, and surface charge optimization, contributing to vaccine consistency and reproducibility.

**Clinical Success and Safety Profiles:** Clinical trials of nanoparticle-based mRNA vaccines have demonstrated high efficacy rates against infectious diseases like COVID-19, underscoring their clinical relevance and translational potential. Safety profiles indicate manageable adverse reactions primarily associated with transient local reactogenicity, highlighting the overall safety of nanoparticle formulations in diverse patient populations.

**Challenges and Mitigation Strategies:** Despite advancements, challenges such as immune activation, biocompatibility concerns, and scalability issues remain significant hurdles in nanoparticle-based mRNA vaccine development. Addressing these challenges requires rigorous preclinical evaluation, the optimization of manufacturing processes, and adherence to stringent regulatory guidelines to ensure vaccine safety, efficacy, and global accessibility.

### 8.2. Implications for Future Vaccine Development

The utilization of nanoparticles in mRNA vaccines not only enhances current vaccine platforms but also sets a foundation for future innovations in vaccine development and personalized medicine. The key implications include the following:

**Advancing Vaccine Design and Technology:** Future vaccine development will benefit from continued advancements in nanoparticle design, enabling tailored vaccine formulations against emerging pathogens and variants. Integrating nanotechnology with mRNA synthesis and delivery systems allows for rapid response capabilities and adaptable vaccine strategies to combat evolving public health threats.

**Precision Medicine and Personalized Vaccination:** Nanoparticle-enhanced mRNA vaccines pave the way for personalized medicine approaches, leveraging mRNA’s programmable nature and nanoparticle versatility to target specific patient populations or disease susceptibilities. This paradigm shift towards precision vaccination offers opportunities for individualized immunization strategies and therapeutic interventions across diverse healthcare contexts.

**Global Health Equity and Accessibility:** Ensuring equitable access to nanoparticle-enhanced mRNA vaccines remains a critical priority in global health initiatives. Collaborative efforts in technology transfer, capacity-building, and regulatory harmonization foster sustainable vaccine production and distribution networks, addressing disparities in vaccine availability and promoting health equity worldwide.

**Innovation in Vaccine Delivery and Adjuvant Systems:** Nanoparticles serve as platforms for innovative vaccine delivery systems and adjuvant strategies, enhancing immune responses and vaccine efficacy. Future research focuses on integrating AI-driven technologies, bioinformatics tools, and predictive modeling to accelerate vaccine discovery, optimize nanoparticle formulations, and enhance therapeutic outcomes in preventive and therapeutic vaccination settings.

So, in terms of ultimate conclusions, nanoparticle technology represents a transformative approach in enhancing the efficacy, stability, and delivery of mRNA vaccines. By overcoming existing challenges and capitalizing on future opportunities, nanoparticle-enhanced mRNA vaccines are poised to revolutionize vaccine development, addressing global health challenges and advancing precision medicine initiatives.
